# Metabolites: fuelling the immune response

**DOI:** 10.1093/cei/uxac053

**Published:** 2022-05-16

**Authors:** Mauro Corrado, Diana Moreira, Nicholas Jones

**Affiliations:** Cologne Excellence Cluster on Cellular Stress Responses in Aging-Associated Diseases (CECAD), University of Cologne, Joseph-Stelzmann-Str. 26, 50931 Cologne, Germany; Institute of Genetics, University of Cologne, Zülpicher Str. 47a, 50674 Cologne, Germany; School of Biochemistry and Immunology, Trinity Biomedical Sciences Institute, Trinity College Dublin, 152-160 Pearse Street, Dublin, 2, Ireland; Institute of Life Science, Swansea University, Swansea, SA2 8PP, UK

Over the last 15 years, our appreciation of the role of metabolism in shaping the innate and adaptive immune systems has profoundly changed. The field, now commonly known as immunometabolism, describes how individual metabolites, metabolic enzymes, and pathways collectively govern immune system plasticity during quiescence and activation [1]. We now understand that cellular metabolism not only underpins the energetic and biosynthetic demands of an immune cell but also plays pivotal roles in intrinsic and extrinsic metabolic regulation, epigenetic mechanisms, and many other immune-orientated functions [2]. Importantly, aberrant immunometabolism is well known to contribute towards a spectrum of human pathologies, with autoimmunity and cancer being two of the most well-researched areas [3, 4]. Moreover, the products of these dysregulated pathways, i.e., individual metabolites have been demonstrated to have multi-faceted immunological roles with certain metabolites exerting context-dependent pro- or anti-inflammatory effects [5, 6]. Indeed, anti-metabolite medication is an important clinical stalwart for conditions such as cancer, infection, and autoimmunity. Methotrexate is utilized in low doses for rheumatoid arthritis and higher doses for cancer acts by inhibiting dihydrofolate reductase and subsequently antagonises one-carbon metabolism. Whilst methotrexate has been around since the 1940s, there is a need for a collective push towards novel innovative therapies exploiting this path. Metabolites, once thought to be no more than waste-products (e.g., lactate), have now been revealed to have important immuno-regulatory roles. One such example, lactate, has been demonstrated to promote regulatory T-cell differentiation in the setting of cancer [7], conversely elevated levels retain autoreactive T cells at the site of inflammation in autoimmunity [8]. Whilst lactate is an excellent, context-dependent example, the functional diversity of many other metabolites throughout central metabolic pathways have now come under scrutiny and are the subject of a vibrant research area. This novel line of thinking has extended far beyond the traditional, one-dimensional metabolic roles or metabolites, with new immune-based roles recently being demonstrated. Key examples of these include the control of immune cell signalling [9], differentiation [10], cellular dysfunction [11], and alterations of migration [8]. This non-exhaustive list evidences the requirement to think ‘outside the box’ and importantly highlights the need for consideration into metabolite exposure both *in vitro* and *in vivo*. Moreover, the recent development of human plasma-like media has ignited a push towards more physiologically relevant experimental models with the attempt to recapitulate the metabolite composition immune cells are exposed to *in vivo*. It is hoped that movement away from traditional experimental models with supraphysiological nutrient levels will better match up *in vitro* and *in vivo* findings, ultimately leading to increased clinical translation and enhanced patient benefit.

As our understanding of immunometabolism, specifically individual metabolites and their underlying roles continues to improve, this can lead to important future discoveries, namely the development of novel metabolism-targeting therapeutics or altered dietary strategies with the aim of improving a range of disease pathologies.

This issue of *Clinical and Experimental Immunology* features the Review Series Metabolites. Here, articles have been collated from worldwide groups with particular research interests in immunometabolism. The scope of topics presented covers a diverse range of pathologies and conditions from autoimmunity, cancer, pregnancy and host protection.

In recent years, multiple studies have addressed autoimmune disorders from an immunometabolic perspective describing how dysregulated metabolism in immune cells can be a trigger of the disorder. In their review, Hanlon et al. [12] discuss the role of metabolites in rheumatoid arthritis (RA). RA is a chronic autoimmune disorder, characterized by progressive degradation of articular cartilage and bone, resulting in functional disability. The combination of altered angiogenesis, hypoxic environment, and immune cell infiltration in the RA joint generate a special inflamed and metabolically altered microenvironment (enriched in lactate, citrate, and succinate) which further exacerbates synovial invasiveness and subsequent deterioration of the cartilage and bone. The authors discuss the potential therapeutic possibilities offered by targeting the accumulation of these metabolites to treat RA and how new approaches based on metabolomics might help stratify patients in terms of outcome and potential responsiveness to treatments.

A second area of great research advances is the study of cell-intrinsic and microenvironmental clues modulating immune function in cancer. Cancer cells alter their metabolism generating a tumour microenvironment where nutrient availability, oxygen and cytokine concentrations dictate an immune-suppressive state which contributes to the progression of cancer and to the absent or limited responsiveness to therapies. This is the classical example of immunotherapy failure in most of solid tumours where the microenvironment is able to block the function of infiltrating immune cells.

Two reviews in this series explore the role of metabolism of immune cells in cancer. In the first one, the role of myeloid-derived suppressor cells (MDSCs) in solid cancers and in particular in non-Hodgkin lymphomas is described by Cioccarelli and Molon [13]. MDSCs are a population of immature immune cells comprising monocytic (M) and polymorphonuclear (PMN) MDSCs responsible for generating an immunosuppressive and tolerogenic tumour microenvironment. They do so by inducing the differentiation of regulatory T (Treg) cells, reducing DC antigen uptake, promoting anti-inflammatory macrophage polarization and repressing cytotoxic T cells. From a metabolic standpoint, MDSCs dampen T-cell effector function by generating extracellular adenosine, depleting amino acids such as arginine, cysteine and tryptophan, and increasing reactive oxygen species (ROS). Targeting MDSCs and limiting their function is a critical goal of multiple ongoing clinical studies, with some of them including targeting metabolic vulnerabilities of MDSCs.

Another strategy aiming at modulating metabolism to improve immune cell function in cancer is directed at dendritic cells (DCs), which sense and capture antigens by pattern recognition receptors and then, after endocytosis and processing, present peptide antigens for T-cell activation. Better understanding of DC function and how it is modulates by metabolism could be central to improve DC-based anti-tumour vaccination which has shown so far limited results. Currivan et al. review the role and impact of different DC subsets in the context of anti-tumour vaccination [14] and the possible metabolic checkpoints to improve critically DC cell function and migration like targeting the integrated stress response (a common pathway activated in response to multiple stress stimuli, including metabolic stress) or core metabolic pathway such as the mevalonate pathway.

Systemic metabolic alterations can also impact directly immune cell function. Metabolic inflammation is a chronic low-grade inflammatory state common in obese individuals and is associated with the development of type 2 diabetes (T2D), non-alcoholic fatty liver disease (NAFLD), and atherosclerosis. In the aforementioned conditions, systemic metabolic alterations include cholesterol metabolism dysregulation and an increase in circulating cholesterol which is able to directly affect immune cell function. Pinzon Grimaldos et al. [15] in their review focus their attention exactly at the crossroad of systemic and cellular metabolism describing how cholesterol metabolism alterations modulates the immunosuppressive capacity of regulatory T cells, which have been described as critical sensors of general metabolism due to their high sensitivity to alteration in nutrient concentration in the extracellular environment. The cholesterol-Treg-inflammation axis generates a negative feedback loop which exacerbates the pathology and the systemic inflammatory state of the affected individual.

Following the same thread of crosstalk between immune function and systemic metabolism Rees et al. [16] discuss how pregnancy modulates both. With pregnancy, maternal physiological changes are necessary to supply nutrient to the foetus including progressive shift from lipid accumulation to lipolysis and from glucose tolerance to insulin resistance. These metabolic changes are paralleled by immunological alterations to generate a tolerogenic state and prevent maternal rejection of the foetus. Interestingly, all these adaptations have a broader effect modulating and reducing the symptoms of autoimmune disorders like rheumatoid arthritis or multiple sclerosis in pregnant women. In this context, the authors discuss how obesity plays a critical role exacerbating inflammation and being linked to adverse outcomes during pregnancy.

In the last review of the series, Gonçalves et al. [17] bring us back to infections and specifically to fungal infection. They represent a major health issue with over 1.5 million deaths each year world-wide affecting healthy and immune-compromised individuals and being an important comorbidity factor when they occur together with viral pneumonia (during influenza or COVID-19). Nevertheless, despite the progress in diagnosis and treatment, severe fungal infections are still a challenge to clinically manage. In this review, the authors discuss the metabolic regulation of the host–fungus interaction and how trained immunity might be exploited as a therapeutic strategy to improve immune response fungal infections. This approach might bring a change in the way we treat this type of infection shifting our attention from targeting the pathogen to harnessing the host metabolism while also limiting the development of unwanted resistance to antifungals.

With this review series we cover the most exciting advances in the field of immunometabolism, while also emphasizing some new line of research where better understanding of immunometabolic crosstalk is central to the development of new diagnosis and therapeutic strategies.

## Data Availability

Not applicable.
