# Correction to: Metabolites: fuelling the immune response

**DOI:** 10.1093/cei/uxac081

**Published:** 2022-08-29

This is a correction to: Mauro Corrado, Diana Moreira, Nicholas Jones, Metabolites: fuelling the immune response, Clinical and Experimental Immunology, Volume 208, Issue 2, May 2022, Pages 129–131, https://doi.org/10.1093/cei/uxac053

In the originally published version of this manuscript, the authors’ names were listed backwards with surnames first and first names last.

This error has been corrected online.

